# Seroepidemiologic Survey of Crimean-Congo Hemorrhagic Fever Virus in Selected Risk Groups, South Africa

**DOI:** 10.3201/eid2407.172096

**Published:** 2018-07

**Authors:** Sabeehah Vawda, Dominique Goedhals, Phillip Armand Bester, Felicity Burt

**Affiliations:** National Health Laboratory Service/University of the Free State, Bloemfontein, South Africa

**Keywords:** Crimean-Congo hemorrhagic fever virus, serosurveillance, South Africa, hemorrhagic virus, viruses, risk factors, vector-borne infections, ticks

## Abstract

Crimean Congo hemorrhagic fever virus (CCHFV) is endemic in South Africa, but whether mild undiagnosed cases occur is unclear. In a seroepidemiologic survey, only 2 of 387 adults considered at risk because of occupational or recreational activities had evidence of previous infection. Seroprevalence in South Africa remains low within the groups investigated.

Crimean-Congo hemorrhagic fever virus (CCHFV; family *Nairoviridae*, genus *Orthonairovirus*) is a tickborne virus that causes human disease (*1*). Humans can be infected through the bite of an infected tick, squashing of an infected tick, or contact with blood or tissues of infected humans or animals. Farmers, herders, veterinarians, hunters, abattoir workers, and persons engaged in informal slaughtering are thus at an increased risk (*2*). 

CCHFV is endemic to Africa, the Middle East, Asia, and southeastern Europe (*2*). Its seroprevalence differs geographically between and within regions. In Greece, CCHFV seroprevalence among various prefectures ranges from 0% to 27.5% (*3*). In Turkey, seroprevalence ranges from 10% to 19.6%, with estimates of 88% subclinical infections (*4*,*5*). Studies among high-risk populations in Iran (*6*) and Oman (*7*) documented seroprevalences of ≈12% and 26.2%, respectively. The factors responsible for subclinical infections are unknown but have been suggested to include differences in host immune responses, viral load, and virus pathogenicity.

In South Africa, surveillance studies found a high prevalence of CCHFV in adult *Hyalomma* ticks and high antibody prevalence in wild and domestic animals (*8*). Two studies among farm workers conducted in the 1980s found a seroprevalence of 1.3%–1.5% (*8*,*9*).

We studied whether the low seroprevalence identified among farm workers reflects that in other high-risk groups. We selected groups on the basis of risk for exposure because of occupational activity, recreational activity, or both and included abattoir workers, horse handlers, recreational hunters, and large animal veterinarians. In South Africa, horse handlers frequently remove ticks from horses, and recreational hunters are exposed to ticks on animals and tissues from animals. The Free State and Northern Cape provinces are farming regions known to have *Hyalomma* ticks. In this study, we therefore aimed to determine the current seroprevalence among healthy persons in selected high-risk groups within CCHFV-endemic provinces of South Africa. 

## The Study

The Health Sciences Research Ethics Committee of the University of the Free State provided ethics approval for this study (HSREC34/2016 and ETOVS152/06). A questionnaire inquiring about demographic and occupational information and possible risk exposure was completed for each volunteer participant. We collected 374 blood samples from volunteers during April 2016–February 2017 and included 13 stored serum samples, collected mainly from large animal veterinarians in 2012.

Specific IgG against CCHFV was detected by using a commercial indirect immunofluorescence assay (IFA) (Crimean-Congo Fever Virus Mosaic 2 IFA; Euroimmun, Lubeck, Germany), according to the manufacturer’s instructions. Each IFA slide contains biochips coated with transfected cells expressing either CCHFV glycoprotein (GP), nucleoprotein (NP), or untransfected cells. We screened serum samples at a dilution of 1:100 and retested positive or undetermined samples using serum diluted 2-fold from 1:100 to 1:800. Samples reacting against CCHFV NP only were retested using 2-fold dilutions from 1:10 to 1:80 for evidence of low reactivity against CCHFV GP. We tested all positive reactors for IgM using IFA.

Most (299 [77.3%]) participants were from the Free State province (Table 1; Figure). Most participants were male (343 [88.6%]), and most resided in urban areas (254 [65.6%]). Ages ranged from 18 to 76 years (median 33 years).

**Table 1:** Study participants at high risk for Crimean-Congo hemorrhagic virus infection, South Africa

Risk group	No. (%) participants, n = 387
Abattoir workers	215 (55.6)
Informal slaughterers	30 (7.8)
Veterinarians	11 (2.8)
Horse handlers	64 (16.5)
Recreational hunters	49 (12.7)
Farmers	12 (3.1)
Other*	6 (1.6)

*Tick exposure, livestock exposure, farm worker.

**Figure:**
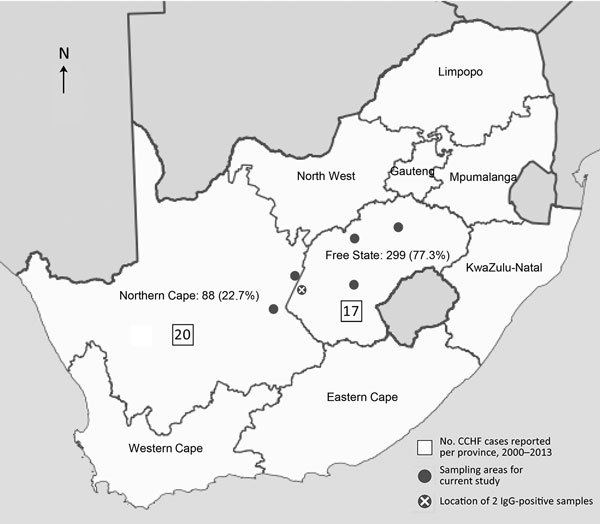
Number and percentage of participants in the Free State Province (177 abattoir workers, 30 informal slaughterers, 11 veterinarians, 32 horse handlers, 46 recreational hunters, 3 other) and Northern Cape Province (38 abattoir workers, 32 horse handlers, 3 recreational hunters, 12 farmers, 3 other) in a seroprevalence study of Crimean-Congo hemorrhagic fever virus in South Africa, April 2016–February 2017.

Abattoir workers formed the largest high-risk group sampled, accounting for 215 (55.6%) of participants. An additional 30 (7.8%) participants were involved in informal slaughtering. Most participants reported multiple potential routes of exposure, either currently or in the past, resulting in considerable overlap among the different groups. A total of 163 (42.1%) participants reported tick exposure; 27 (7%) participants reported an illness after a tick bite or exposure to animal blood or tissue, and 18 (4.7%) reported a confirmed diagnosis of tick-bite fever.

Of the 387 serum samples tested, 2 tested positive for CCHFV IgG. The seropositive samples were collected from men, both 27 years of age, who were abattoir workers at the same abattoir in rural Free State. Both participants had additional potential CCHFV risk exposures, including tick exposure and hunting (Table 2). Neither participant reported any illness after a tick bite or after exposure to animal blood or tissue, and both were healthy at the time the blood was collected.

**Table 2:** Detailed risk exposure for the 2 Crimean-Congo hemorrhagic fever virus–positive study participants, South Africa

Risk exposure	Participant 1	Participant 2
Farmer	No	Yes
Farm worker	Yes	No
Tick exposure	Yes	Yes
Livestock exposure	No	Yes
Hunter	Yes	Yes
Abattoir worker	Yes	Yes
Horse handler	Yes	Yes
Veterinarian	No	No
Veterinary researcher	No	No
Laboratory worker	No	No
Major illness after tick bite	No	No
Major illness after exposure to animal blood or tissue	No	No

IgG-positive samples for both men tested IgM negative, which excluded acute or recent infections. The IgG titers obtained for participant 1 were 1:100 against the NP and 1:80 against the GP antigen. The IgG titer for participant 2 was 1:400 against the NP antigen only. The variation in antibody titers against NP and GP is not unexpected and has been reported previously, although the reason is unknown. Evidence exists of serologic cross reactivity between CCHFV and Hazara virus; however, previous serologic surveys suggest that Hazara virus is not circulating in South Africa (*10*).

CCHFV is considered an emerging virus with potential for spread to areas where *Hyalomma* ticks are present (*2*). In terms of which populations are particularly at risk for infection, a retrospective study in Iran found that 34% of confirmed CCHFV cases were in slaughterhouse workers and 28.5% were in farmers or livestock handlers (*11*). Similarly, a study in Kenya found that 19% of patients with a febrile illness were eventually confirmed to have CCHFV; the highest prevalence (29.3%) occurred among farmers (*12*). Many seroprevalence studies have documented the unanticipated finding of asymptomatic or mild disease. In Greece, the low number of cases of infection with the high seroprevalence has been suggested to indicate circulation of a strain that is potentially of lower virulence (*13*). The use of different serologic methods must be considered in comparing the results of surveys. However, a frequently used assay in recent studies is the commercially available ELISA (Vektor-Best, Novosibirsk, Russia) that, when compared with IFA, showed reasonably comparable sensitivities of 80.4% and 86.1%, respectively.

Since 1981, when CCHFV was first identified in South Africa, sporadic cases have been reported mainly from the country’s central farming areas. The principal vectors associated with transmission, *H. truncatum* and *H. rufipes* ticks, are widely distributed throughout South Africa but are most numerous in the interior of the country, where prevalence of CCHF antibody in cattle serum is high; up to 96% of cattle serum tested in some herds was positive. CCHFV was isolated in ≈20% of tick pools, representing both tick species, collected in the North West province (*10*,*14*). The Free State and Northern Cape provinces are considered CCHFV-endemic regions. During 1981–2013, a total of 192 CCHFV cases were laboratory confirmed in South Africa; 54 laboratory-confirmed cases were documented during January 2000–August 2013. Of these, 17 (31.5%) were from the Free State and 20 (37%) from the Northern Cape (Figure) (*15*).

## Conclusions

Our seroprevalence results were similar to those obtained 30 years ago among farm workers (*8*,*9*), indicating that, even within high-risk groups, CCHFV remains uncommon in South Africa. The number of participants was low but focused on selected high-risk populations.

The 2 participants with CCHFV IgG tested negative for CCHFV IgM and recalled no previous illness resembling severe Crimean-Congo hemorrhagic fever, which might hint at possible mild CCHF in South Africa. However, in view of documented widespread CCHFV and antibodies in ticks and animals, respectively, in South Africa (*8*), more widespread mild infection would be anticipated. Our study conducted among groups at high risk for CCHFV in the endemic regions of Free State and Northern Cape provinces found that the seroprevalence of the virus remains low as previously shown, despite multiple potential routes of exposure in the cohort.
